# Loss of WNT2B Increases Tumor Burden and Malignant Features in Colorectal Cancer

**DOI:** 10.34133/cancomm.0036

**Published:** 2026-06-24

**Authors:** Luiz Fernando Silva Oliveira, Yu-Syuan Wu, Sathuwarman Raveenthiraraj, Jaedeok Kwon, Venkata S. Dasuri, Comfort Adegboye, Juan Putra, Jorge O. Munera, Diana L. Carlone, David T. Breault, Amy E. O’Connell

**Affiliations:** ^1^ Division of Newborn Medicine, Boston Children’s Hospital, Boston, MA 02115, USA.; ^2^ Department of Pathology, Boston Children’s Hospital, Boston, MA 02115, USA.; ^3^ Department of Regenerative Medicine and Cell Biology, Hollings Cancer Center, Medical University of South Carolina, Charleston, SC 29425, USA.; ^4^ Department of Pediatrics, Harvard Medical School, Boston, MA 02115, USA.; ^5^ Division of Endocrinology, Boston Children’s Hospital, Boston, MA 02115, USA.; ^6^ Harvard Stem Cell Institute, Cambridge, MA 02138, USA.; ^7^ Manton Center for Orphan Disease Research, Boston Children’s Hospital, Boston, MA 02115, USA.

## Introduction

Hyperactivation of the Wingless-related integration site (WNT) signaling pathways drives nearly all cases of colorectal cancer (CRC) [1–3]. However, the role of individual WNT proteins in CRC development remains poorly understood. Nineteen distinct secreted proteins, known as WNT ligands, have been identified in humans, including WNT2B [4]. We previously demonstrated that WNT2B loss-of-function (LOF) is associated with abnormal intestinal morphology in humans and decreased expression of intestinal stem cell markers in humans and mice [5–7]. Clinically, WNT2B LOF causes severe congenital enteropathy in humans [5]. Meanwhile, *Wnt2b* knockout (KO) mice exhibited enhanced susceptibility to early injury in experimental acute colitis, driven by increased immune cell recruitment and proinflammatory cytokine production [7]. Another research group has recently reported that human WNT2B LOF also predisposes affected subjects to gastrointestinal dysplasia with age [8]. Here, we examine how WNT2B is associated with colorectal tumor burden and histopathological features in human datasets and how WNT2B LOF impacts mouse models of inflammation-associated and sporadic tumorigenesis.

**Fig. 1.**
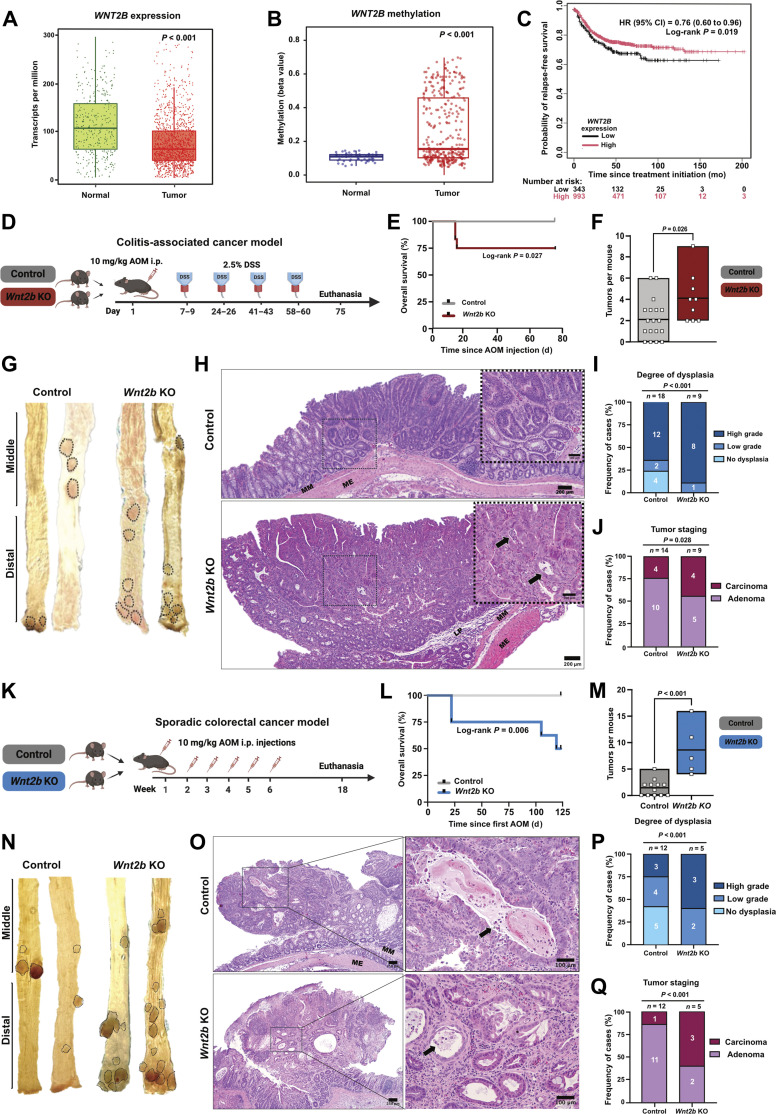
WNT2B loss predicts a worse prognosis in colorectal cancer in mice and humans. (A) *WNT2B* expression levels in normal (*n* = 377) and colon cancer samples (*n* = 1,450) from TCGA’s colon adenocarcinoma dataset. (B) Methylation levels of the *WNT2B* gene in CRC. (C) Kaplan–Meier curves show the association between high (red) and low (black) *WNT2B* expression levels and relapse-free survival of patients from all stages, calculated by a Mantel–Cox log-rank test (*P* = 0.023). (D) Adapted azoxymethane/dextran sodium sulfate (AOM/DSS) protocol with sex- and age-matched controls (*n* = 18) and *Wnt2b* KO (*n* = 12) mice receiving 10 mg/kg of AOM on day 1, followed by 4 cycles of 2.5% DSS in drinking water for 3 d, with 2-week recovery periods between each cycle. (E) Kaplan–Meier survival analysis of control (*n* = 18) and *Wnt2b* KO (*n* = 12) mice following AOM/DSS treatment using a log-rank test (*P* = 0.027). (F) Box plots showing group means ± SD of tumor burden per mouse, comparing control and *Wnt2b* KO mice with a 2-tailed Mann–Whitney *U* test (*P* = 0.026). (G) Representative macroscopic view of the intestines from control and *Wnt2b* KO mice, highlighting tumors throughout the colon. (H) Representative H&E-stained sections. The top panel shows a representative high-grade dysplasia adenoma from control mice, with an inset highlighting the dysplastic glandular structures. The bottom panel displays a representative intraepithelial adenocarcinoma from *Wnt2b* KO mice, with an inset highlighting desmoplastic stroma and dirty necrosis (arrow). Scale bars 200 and 100 μm, respectively. H&E staining was used to assess (I) the degree of dysplasia and (J) tumor staging in control and *Wnt2b* KO mice, and comparisons were made using the chi-square test. (K) Sporadic CRC model with sex- and age-matched controls (*n* = 12) and *Wnt2b* KO (*n* = 8) mice receiving 6 weekly intraperitoneal (i.p.) injections of 10 mg/kg AOM, followed by a 12-week recovery period under standard conditions until the tissue harvesting. (L) Kaplan–Meier survival analysis of control (*n* = 12) and *Wnt2b* KO (*n* = 8) mice after AOM injections, using a log-rank test (*P* = 0.006). (M) Floating bars showing group means ± SD of tumor multiplicity per colon in control (*n* = 12) and *Wnt2b* KO (*n* = 5) mice, calculated by a 2-tailed Mann–Whitney *U* test (*P* < 0.001). (N) Macroscopic view of the distal colon in control and *Wnt2b* KO mice, highlighting tumors (dotted lines). (O) Representative H&E-stained intraepithelial adenocarcinoma in control (top panel) and *Wnt2b* KO mice (bottom panel). Scale bars 250 μm. Enlarged images show differences in glandular secretions and immune cell content, with heterogeneous epithelial and stromal morphologies and dirty necrosis (arrow). Scale bars 100 μm. H&E staining was used to evaluate (P) the degree of dysplasia and (Q) tumor staging. Control (*n* = 12) and *Wnt2b* KO (*n* = 5) groups were compared using the chi-square test. AOM, azoxymethane; CI, confidence interval; CRC, colorectal cancer; DSS, dextran sodium sulfate; H&E, hematoxylin and eosin; HR, hazard ratio; KO, knockout; LP, lamina propria; MM, muscularis mucosae; SM, submucosa; ME, muscularis externa; SD, standard deviation; TCGA, The Cancer Genome Atlas; WNT2B, Wnt family member 2B.

We first analyzed data from The Cancer Genome Atlas and observed significantly lower *WNT2B* expression in CRC compared with normal tissue (Fig. 1A). Tumors exhibited increased *WNT2B* methylation (Fig. 1B), which may contribute to reduced transcript levels (Fig. S1A). *WNT2B* expression did not differ across the 4 main disease stages (Fig. S1B) and was not associated with age, sex, race, tumor site, histologic subtype, or overall pathological stage (Table S1). However, low *WNT2B* expression was associated with reduced relapse-free survival (Fig. 1C), particularly in early disease stages (Fig. S1C and D). Genomic alterations in *WNT2B* were observed in 33% of analyzed samples and were associated with worse overall survival than in tumors carrying the consensus sequence (Fig. S1E and F). Interestingly, *WNT2B* gains or amplifications were uncommon (3%) compared to other WNT ligands (Fig. S1G). At the molecular level, *WNT2B*-low tumors were significantly enriched for *KRAS* (Kirsten rat sarcoma viral oncogene homolog) and *BRAF* (B-Raf proto-oncogene, serine/threonine kinase) mutations (Table S2), as well as MSI-H (microsatellite instability-high) status across both MSIsensor and MANTIS (Microsatellite Analysis for Normal-Tumor InStability) metrics. These tumors also showed higher hypoxia-related gene expression scores in both the Winter and Buffa signatures (Table S1). Importantly, these molecular features were observed independently of baseline clinicopathologic characteristics. Together, these data suggest that reduced *WNT2B* expression reflects a biologically distinct molecular subset that may contribute to its association with poorer survival, independent of anatomic site and pathologic stage.

Next, we established an azoxymethane/dextran sodium sulfate (AOM/DSS) model of colitis-associated cancer (Fig. 1D and Supplementary Methods). *Wnt2b* KO mice exhibited greater weight loss and intestinal bleeding than their control littermates, resulting in worsened overall disease severity and reduced survival (Fig. 1E and Fig. S2A to F). *Wnt2b* KO mice developed more tumors overall, especially in the distal colon (Fig. 1F and G and Fig. S2G and H). Histopathological assessment revealed larger tumors and more extensive mucosal injury in *Wnt2b* KO mice than in control littermates (Fig. S3A to G). Although inflammatory mediators *TNFα* (tumor necrosis factor alpha), *IL-1β* (interleukin-1 beta), and *IL-6* (interleukin-6) were induced in both groups following AOM/DSS treatment (Fig. S3H to J), *IL-6* expression was significantly higher in *Wnt2b* KO mice compared to the control group (Fig. S3J). Notably, while 22% of control animals showed no dysplasia or tumor formation, colorectal lesions in *Wnt2b* KO mice were ubiquitous and were frequently classified as high-grade dysplasia or carcinoma (Fig. 1H and I), with carcinomas accounting for a larger fraction of total lesions (Fig. 1J). We did not observe differences in transcript-level expression of classical β-catenin-dependent target genes (Fig. S4). However, canonical WNT/β-catenin signaling activity was not directly assessed, and subtle or cell-type-specific canonical effects cannot be excluded. Together, these findings indicate that WNT2B deficiency exacerbates inflammation-driven colorectal tumorigenesis, but they do not establish the precise signaling mechanism. While our data are consistent with recent reports of non-canonical [9] and inflammation-associated roles for WNT2B [5–8], dedicated mechanistic studies will be required to formally define the pathways that contribute to this phenotype.

Because the colitis-associated cancer model combines mutagenesis (AOM) and inflammatory injury (DSS), we next asked whether a similar tumor-promoting phenotype would be observed in a mutagenesis-only model of sporadic CRC (Fig. 1K and Supplementary Methods). In this setting, overall body weight was comparable between the 2 groups (Fig. S5A and B). However, survival remained reduced in *Wnt2b* KO mice, with 63% reaching humane endpoints due to rectal prolapse (Fig. 1L and Fig. S5C to E). Since rectal prolapse, when accompanied by weight loss, has been described as a surrogate marker of distal tumor burden in sporadic CRC mouse models, it was not used as an exclusion criterion in our study [10]. All animals that completed the 12-week endpoint following AOM exposure, including those that met humane endpoint criteria on the final week, were included in the formal analyses (Table S3). A higher incidence of rectal prolapse and greater body weight changes were observed in females compared to males (Fig. S5E to I). Colon length and spleen weight were comparable between the 2 groups (Fig. S5J and K). Although the study was not powered to formally evaluate sex-specific mechanisms, these findings suggest that WNT2B signaling may interact with sex-dependent immune or hormonal pathways that regulate epithelial repair, warranting further investigation. Tumor burden was increased in *Wnt2b* KO mice, as all animals developed at least 3 tumors, whereas this occurred in only 46% of control littermates (Fig. 1M and Fig. S6A). Tumors were again enriched in the distal colon and were associated with larger hyperplastic and dysplastic tumor areas in *Wnt2b* KO mice (Fig. 1N and Fig. S6B to G). Consistently, tumors in *Wnt2b* KO mice exhibited more malignant features than those in controls (Fig. 1O to Q), indicating that WNT2B LOF increases the susceptibility to tumor development even in the absence of induced inflammation.

Together, these data demonstrate that loss of WNT2B increases tumor burden and promotes progression toward higher-grade lesions, with consistent effects observed in both inflammatory and non-inflammatory tumor models. While we did not perform temporal analyses to formally demonstrate accelerated kinetics of the dysplasia-to-carcinoma transition, the increased frequency of advanced lesions suggests that WNT2B deficiency facilitates malignant progression rather than affecting tumor initiation alone. Cross-species analyses integrating human genomic data with 2 independent mouse models support an association between reduced *WNT2B* expression and poorer clinical outcomes in CRC. Importantly, while our findings define robust and reproducible phenotypic relationships, they do not establish the cellular source or signaling mechanisms underlying tumor susceptibility. Determining whether these effects arise from epithelial, stromal, or immune compartments, and whether restoring WNT2B activity within defined cell populations can modify disease trajectory, represents important future directions for this work.

## Ethical Approval

All animal experiments were approved by Boston Children’s Hospital’s Institutional Animal Care and Use Committee (IACUC) under protocol no. 00001978.

## Data Availability

The data generated in this study are available within the article and its supplementary data files. The raw data are also available by direct request via email to the corresponding author.
