# Supplementary material for: Loss of WNT2B Increases Tumor Burden and Malignant Features in Colorectal Cancer
Source: Cancer Commun (Lond). 2026 Jun 24;46:0036. doi: 10.34133/cancomm.0036 (PMC13291482; doi:10.34133/cancomm.0036)
Supplement: Supplementary 1 — Tables S1 to S6 Figs. S1 to S7 [file cancomm.0036.f1.docx]

**Supplementary Materials and Methods for**

Loss of WNT2B Increases Tumor Burden and Malignant Features in Colorectal Cancer

Luiz Fernando Silva Oliveira^1, *^, Yu-Syuan Wu^1^, Sathuwarman Raveenthiraraj^1^, Jaedeok Kwon^1^, Venkata S. Dasuri^1^, Comfort Adegboye^1^, Juan Putra^2^, Jorge O. Munera^3^, Diana L. Carlone^4, 5, 6^, David T. Breault^4, 5, 6^, Amy E. O’Connell^1, 4, 7, *^

^1^ Division of Newborn Medicine, Boston Children’s Hospital, Boston, Massachusetts, 02115, USA.

^2^ Department of Pathology, Boston Children’s Hospital, Boston, Massachusetts, 02115, USA.

^3^ Department of Regenerative Medicine and Cell Biology, Hollings Cancer Center, Medical University of South Carolina, Charleston, South Carolina, 29425, USA.

^4^ Department of Pediatrics, Harvard Medical School, Boston, Massachusetts, 02115, USA.

^5^ Division of Endocrinology, Boston Children’s Hospital, Boston, Massachusetts, 02115, USA.

^6^ Harvard Stem Cell Institute, Cambridge, Massachusetts, 02138, USA.

^7^ Manton Center for Orphan Disease Research, Boston Children’s Hospital, Boston, Massachusetts, 02115, USA.

^*^Corresponding authors:

Luiz Fernando Silva Oliveira, Division of Newborn Medicine, Boston Children’s Hospital. Enders 9, 300 Longwood Ave, Boston, Massachusetts, 02115, USA. E-mail: [fernando.oliveira@childrens.harvard.edu](mailto:fernando.oliveira@childrens.harvard.edu)

Amy E. O’Connell, Division of Newborn Medicine, Boston Children’s Hospital. Enders 9, 300 Longwood Ave, Boston, Massachusetts, 02115, USA. E-mail: [amy.oconnell@childrens.harvard.edu](mailto:amy.oconnell@childrens.harvard.edu)

**Rigor and Reproducibility**

Experimental data from mouse experiments represent a minimum of six biological replicates, which, based on our previous calculations using an alpha of 0.05, gives adequate power to detect meaningful differences between control littermates and Wingless-related integration site (Wnt) family member 2b knock-out (*Wnt2b* KO) mice.

**Reagents**

**Supplementary Table S4** lists all reagents used in this study, including manufacturer, catalog number, antibody clone, and concentration.

**Mice**

*Wnt2b* KO mice and control littermates were generated as previously reported [1-3]. No significant differences were found in *Wnt2b* expression or experimental outcomes between wild-type (WT) and heterozygous littermates [1]. Therefore, *Wnt2b* WT and heterozygous individuals were combined into the control group throughout the manuscript. All mice were maintained in micro-isolator cages with free access to food and water under a 12-hour light/12-hour dark cycle at the Animal Resources at Boston Children's Hospital (ARCH). Genotyping was performed by Reverse Transcription Polymerase Chain Reaction (RT-PCR) to confirm *Wnt2b* ablation prior to inclusion in experimental cohorts (**Supplementary Figure S7A**). Tissue-specific deletion was further validated by quantitative RT-PCR (qRT-PCR) to confirm the absence of *Wnt2b* transcript in the baseline colon (**Supplementary Figure S7B**) and prior to downstream experimental analyses (**Supplementary Figure S7C**).

**Colitis-Associated Cancer Model**

We adapted the well-established azoxymethane/dextran sodium sulfate (AOM/DSS) model of colitis-associated cancer (CAC), given our previous study showing that *Wnt2b* KO mice are more sensitive to DSS [1] and would likely die from the treatment if we followed the standard 5–7-day colitis cycle [4, 5]. However, we included an extra DSS cycle to facilitate tumor development. On experimental day 1, male and female control and *Wnt2b KO* mice aged 8-12 weeks received a single intraperitoneal (i.p.) injection of AOM (Sigma-Aldrich) (dose = 10 mg/kg of body weight). Five days later, we treated the mice with 2.5% DSS (MPBio) in their drinking water for three days. The mice were then allowed to recover for sixteen days, and this regimen was repeated four times. Weight loss, stool consistency, and rectal bleeding were scored daily to generate a semiquantitative assessment of clinical colitis [4, 5]. See **Supplementary Table S5** for detailed Disease Activity Index (DAI) scores. Mice were euthanized on day 75 or if they met criteria for early euthanasia, which included extreme distress, hunching, rectal prolapse, or if they lost more than 20% of their initial body weight.

**Sporadic Colorectal Cancer Model**

Male and female *Wnt2b* control and *Wnt2b* KO mice aged 8-12 weeks were treated weekly with an i.p. injection of 10 mg/kg AOM for six consecutive weeks to mimic sporadic colorectal cancer [6, 7]. Changes in body weight, feces consistency, rectal bleeding, or any signs of distress or behavioral changes were recorded daily. Mice were euthanized 12 weeks after the first AOM injection or sooner whenever they presented extreme distress and met criteria for early euthanasia, which included hunching, rectal prolapse, or losing more than 20% of their initial body weight.

**Histology**

After euthanasia, colons were removed, measured, cleaned, and opened longitudinally. The tissue was then -rolled, placed in fixation cassettes, and fixed for 24 hours in 10% Neutral-Buffered Formalin at room temperature (RT). Following routine histology processing, the formalin-fixed paraffin-embedded (FFPE) tissue samples were sectioned at 5 µm depth for Hematoxylin and Eosin (H&E) or Periodic Acid Schiff (PAS) staining. Histologic assessment of colitis, dysplasia, and tumor staging was performed by a gastroenterology pathologist blinded to the study design. H&E and PAS stains were performed to evaluate for mucosal and submucosal inflammatory cell infiltrates, epithelial abnormalities (crypt hyperplasia and goblet cell loss), crypt loss, ulceration, and crypt abscess. Dysplasia was classified as low-grade or high-grade based on nuclear atypia (polarization, stratification, and chromatin), epithelial differentiation, and abnormal growth patterns [8, 9]. Detailed histopathological scores are described in **Supplementary Table S6**.

**Digital Image Analysis**

Whole-slide image analysis of H&E-stained Swiss-rolls from mouse colons was performed using QuPath (version 0.6.0) [10]. Tile scan images were obtained at 20X magnification using an EVOS FL Auto Imaging System (Thermo Fisher Scientific). Images were opened, and tumor regions previously identified in the histopathological analysis were manually annotated using the freehand selection method, as previously described [11]. Annotations were stored as region-of-interest (ROI) objects. Using established criteria, tumors were then classified by anatomical location (proximal, middle, and distal) and stage (low-grade and high-grade adenoma, or adenocarcinoma) [8, 9]. The tumor area (µm²) was calculated in QuPath, and the measurement data were exported. Area values were converted to mm² in Microsoft Excel using a custom macro [10, 11].

**RNA Purification and Real-Time Polymerase Chain Reaction**

Distal colon samples (1 cm) were obtained from untreated controls and AOM/DSS-treated animals. Samples were digested with TRIzol™ Reagent (Invitrogen), and RNA was purified using the RNeasy Mini Kit (Qiagen), according to the manufacturer’s instructions. RNA was quantified with a NanoDrop (Invitrogen) and reverse transcribed into cDNA using a high-capacity cDNA reverse transcription kit (Thermo-Fisher) per the manufacturer’s instructions. Gene expression was analyzed by real-time polymerase chain reaction (PCR) on a QuantStudio6 Flex (Thermo Fisher) using TaqMan qPCR Master Mix and specific primers, as specified in **Supplementary Table S4**. Technical duplicates or triplicates were implemented to account for technical variability, with 18S as an internal control. Fold change was calculated using the ΔΔCt method.

**Public Database and Bioinformatic Analyses**

To evaluate the effects of *WNT2B* loss of expression in colorectal cancer (CRC), we analyzed the Colorectal Adenocarcinoma (TCGA, PanCancer Atlas) cohort using cBioPortal for Cancer Genomics (<https://www.cbioportal.org/>) [12]. This dataset included 594 patients with available genomic and clinicopathologic data. WNT2B expression groups were defined using mRNA expression z-scores relative to all samples (log RNA-Seq V2 RSEM), with high (≥ 0.07) and low (≤ 0.06) groups established based on the cohort median expression value within cBioPortal. Associations between WNT2B expression and clinicopathologic variables included pathologic stage, tumor location, microsatellite instability (MSI) status, somatic mutations, and gene copy number alterations. WNT2B expression profiles in normal and colorectal tumor samples were analyzed using the TNMplot (<https://tnmplot.com/analysis/>) [13] and Gene Expression Profiling Interactive Analysis (GEPIA2) (<http://gepia2.cancer-pku.cn/>) [14]. The association between WNT2B expression and patient survival was assessed using the Kaplan–Meier Plotter platform (KM Plotter) [15]. WNT2B methylation status and its correlation with gene expression and survival outcomes were evaluated using the Gene Set Cancer Analysis (GSCA) database (<https://guolab.wchscu.cn/GSCA/>) [16]. For analyses involving multiple related comparisons, p-values were adjusted using the Benjamini–Hochberg false discovery rate (FDR) correction or Bonferroni adjustment, as appropriate.

**Statistical Analyses**

Statistical analyses were performed using GraphPad Prism 10 or R software. All data were tested for normality using the Shapiro-Wilk test, with *P* - values < 0.05 considered statistically significant for all downstream analyses. Tumor incidence and sizes between Control and *Wnt2b KO* mice were compared by two-sided Fisher’s exact test and unpaired two-tailed t-test, respectively. Gene expression was compared using two-way ANOVA with Tukey’s post hoc test. Kaplan-Meier survival analysis was performed to assess survival in mouse models, with significance determined by the log-rank test.

**Supplementary Tables**

**Supplementary Table S1. Association between *WNT2B* expression and clinicopathological variables, and transcriptomic-based assessment of microsatellite instability and hypoxia scores.**

| Clinical Attributes | *WNT2B*  High  (*n* = 296) | *WNT2B*  Low  (*n* = 296) | Analysis | | |
| --- | --- | --- | --- | --- | --- |
|  | Cases (%) | Cases (%) | Method | *P*-value | FDR |
| Age at diagnosis, years | | | Wilcoxon Test | 0.906 | 0.954 |
| <60 | 84/294 (29%) | 85/294 (29%) |  | | |
| ≥60 | 210/294 (71%) | 209/294 (71%) |  | | |
| Sex | | | Chi-squared Test | 0.742 | 0.872 |
| Female | 123/293 (42%) | 126/293 (43%) |  | | |
| Male | 170/293 (58%) | 167/293 (57%) |  | | |
| Race category | | | Chi-squared Test | 0.526 | 0.754 |
| Asian | 4/174 (2%) | 11/172 (6%) |  | | |
| American Indian or Alaska Native | 1/174 (1%) | 0/172 (0%) |  | | |
| Black or African American | 41/174 (24%) | 37/172 (22%) |  | | |
| White | 124/174 (71%) | 124/172 (72%) |  | | |
| Pathologic stage | | | Chi-squared Test | 0.281 | 0.552 |
| Stage I | 47/288 (16%) | 46/294 (16%) |  |  |  |
| Stage II | 97/288 (34%) | 104/294 (35%) |  |  |  |
| Stage III | 94/288 (33%) | 92/294 (31%) |  |  |  |
| Stage IV | 50/288 (17%) | 52/294 (18%) |  |  |  |
| Tumor Site | | | Chi-squared Test | 0.108 | 0.336 |
| Colon | 232/294 (79%) | 232/297 (78%) |  |  |  |
| Rectum | 62/294 (21%) | 65/297 (22%) |  |  |  |
| Histological type |  |  | Chi-squared Test | 0.361 | 0.617 |
| Conventional Adenocarcinoma | 239/296 (81%) | 246/296 (83%) |  | | |
| Mucinous Adenocarcinoma | 57/296 (19%) | 50/296 (17%) |  | | |
| MSIsensor score | | | Wilcoxon Test | < 0.001 | < 0.001 |
| MSS | 268/295 (91%) | 224/294 (76%) |  | | |
| MSI-I | 5/295 (2%) | 7/294 (2%) |  | | |
| MSI-H | 22/295 (7%) | 63/294 (21%) |  | | |
| MANTIS score | | | Wilcoxon Test | < 0.001 | < 0.001 |
| MSI-H | 111/282 (39%) | 137/279 (49%) |  | | |
| MSS | 171/282 (61%) | 142/279 (51%) |  | | |
| Winter hypoxia score | | | Wilcoxon Test | < 0.001 | < 0.001 |
| Median (IQR) | 14 (24–0) | 20 (28–10) |  | | |
| Hypoxia-Low^a^ | 92/166 (55%) | 81/199 (41%) |  | | |
| Hypoxia-High^a^ | 74/166 (45%) | 118/199 (59%) |  | | |
| Buffa hypoxia Score |  | | Wilcoxon Test | < 0.001 | < 0.001 |
| Median (IQR) | 15 (25–5) | 23 (33–15) |  | | |
| Hypoxia-Low^b^ | 103/166 (62%) | 80/199 (40%) |  | | |
| Hypoxia-High^b^ | 63/166 (38%) | 119/199 (60%) |  | | |

^a^Winter Hypoxia Scores and ^b^Buffa Hypoxia Scores were dichotomized using the median score observed in WNT2B-LOW tumors, thereby defining hypoxia categories relative to the WNT2B-LOW reference distribution. **Abbreviations:** WNT2B, Wingless-type MMTV integration site family member 2B; N, number of samples; IQR, interquartile range; MSS, microsatellite stable; MSI, microsatellite instability; MSI-I, microsatellite instability–intermediate; MSI-H, microsatellite instability–high; MSIsensor, Microsatellite Instability Sensor algorithm; MANTIS, Microsatellite Analysis for Normal-Tumor InStability algorithm; FDR, false discovery rate; Wilcoxon test, Wilcoxon rank-sum test; Chi-squared test, Pearson’s chi-squared test.

**Supplementary Table S2. Association between *WNT2B* expression and genomic alterations.**

| Gene | *WNT2B* | | Analysis | | | | |
| --- | --- | --- | --- | --- | --- | --- | --- |
|  | High  (*n* = 296) | Low  (*n* = 296) |  |  |  |  |  |
|  | Cases (%) | Cases (%) | OR | 95% CI | Method | *P*-value | FDR |
| *KRAS* | 97 (36%) | 12 (46%) | 1.48 | 1.04–2.11 | Chi-square | 0.029 | 0.048 |
| *BRAF* | 17 (6%) | 45 (17%) | 3.01 | 1.68–5.38 | Fisher’s exact | < 0.001 | 0.001 |
| *TP53* | 166 (62%) | 146 (55%) | 0.75 | 0.54–1.05 | Chi-square | 0.094 | 0.118 |
| *APC* | 193 (73%) | 192 (73%) | 1.00 | 0.68–1.48 | Chi-square | 0.999 | 0.999 |
| *CTNNB1* | 10 (4%) | 23 (9%) | 2.44 | 1.11–5.38 | Fisher’s exact | 0.026 | 0.065 |

**Abbreviations:** *WNT2B*, Wingless-type MMTV integration site family member 2B; *KRAS*, Kirsten rat sarcoma viral oncogene homolog; *BRAF*, B-Raf proto-oncogene, serine/threonine kinase; *TP53*, tumor protein p53; *APC*, adenomatous polyposis coli; *CTNNB1*, catenin beta 1 (β-catenin); OR, odds ratio; CI, confidence interval; FDR, false discovery rate; n, number of samples; Wilcoxon test, Wilcoxon rank-sum test.

**Supplementary Table S3. Per-animal raw data for the sporadic colorectal cancer mouse model.**

| Group | Sex | Mouse ID | Affected by rectal prolapse | Overall histopathological score | Tumor staging | | | | Total tumor area (mm^2^) |
| --- | --- | --- | --- | --- | --- | --- | --- | --- | --- |
|  |  |  |  |  | **Adenoma** | | **Carcinoma** | **Total** |  |
|  |  |  |  |  | **LGD** | **HGD** |  |  |  |
| Control | Male | 980 | No | 3 | 0 | 0 | 0 | 0 | 0.000 |
|  |  | 981 | No | 7 | 1 | 0 | 0 | 1 | 0.793 |
|  |  | 987 | No | 8 | 0 | 0 | 0 | 0 | 0.000 |
|  |  | 988 | No | 5 | 1 | 1 | 0 | 2 | 3.442 |
|  |  | 989 | No | 3 | 2 | 0 | 0 | 2 | 0.162 |
|  |  | 990 | No | 5 | 0 | 0 | 0 | 0 | 0.000 |
|  |  | 995 | No | 6 | 0 | 0 | 0 | 0 | 0.000 |
|  | Female | 976 | No | 8 | 1 | 0 | 0 | 1 | 0.051 |
|  |  | 977 | No | 4 | 0 | 0 | 0 | 0 | 0.000 |
|  |  | 978 | No | 5 | 2 | 0 | 0 | 2 | 0.501 |
|  |  | 979 | No | 10 | 2 | 1 | 1 | 4 | 5.155 |
|  |  | 998 | No | 9 | 2 | 1 | 0 | 3 | 1.200 |
| *Wnt2b* KO | Male | 982 | No | 6 | 2 | 0 | 0 | 2 | 0.787 |
|  |  | 993 | No | 11 | 2 | 2 | 3 | 7 | 9.118 |
|  | Female | 991 | No | 10 | 3 | 0 | 0 | 3 | 0.771 |
|  |  | 996 | Yes | 10 | 2 | 1 | 5 | 8 | 11.182 |
|  |  | 997 | Yes | 11 | 3 | 2 | 2 | 7 | 7.696 |
|  | The mice shown below this line died before the 12-week endpoint and were not included in the formal analysis. | | | | | | | | |
|  | Female | 992 | Yes | 6 | 2 | 0 | 0 | 2 | 1.431 |
|  | Male | 986 | Yes | 7 | 3 | 0 | 0 | 3 | 0.811 |
|  |  | 994 | Yes | 13 | 3 | 0 | 0 | 3 | 1.355 |

**Abbreviations:** *Wnt2b,* Wnt family member 2b; LGD, low-grade dysplasia; HGD, high-grade dysplasia; KO, knockout.

**Supplementary Table S4. Key Resources.**

| **Reagents** | **Source** | **Catalog number** |
| --- | --- | --- |
| *Chemicals* | | |
| Azoxymethane (AOM) | Sigma-Aldrich | A5486 |
| Dextran Sodium Sulfate (DSS) | MP Biomedicals | 160110 |
| 10% Neutral Buffered Formalin Solution | Sigma-Aldrich | HT501128 |
| 1X Phosphate Buffered Solution, pH 7.4 | Gibco | 10010023 |
| TRIzol™ Reagent | Invitrogen | 15596018 |
| 28G Insulin Syringe 1ml | BD | 329424 |
| Ethyl Alcohol 200 Proof | Pharmco-Aaper | 111000200CSGL |
| *Critical Commercial Assays* | | |
| RNeasy Mini Kit | QIAGEN | 74104 |
| High-Capacity cDNA Reverse Transcription Kit | ThermoFisher | 4368813 |
| TaqMan™ Universal PCR Master Mix | ThermoFisher | 4364338 |
| *Oligonucleotides* | | |
| *18S* | ThermoFisher | Mm03928990_g1 |
| *Wnt2b* | ThermoFisher | Mm00437330_m1 |
| *Lef1* | ThermoFisher | Mm00550265_m1 |
| *Axin2* | ThermoFisher | Mm01265779_m1 |
| *Lgr5* | ThermoFisher | Mm00438890_m1 |
| *Myc* | ThermoFisher | Mm00487804_m1 |
| *IL-6* | ThermoFisher | Mm00446190_m1 |
| *TNFa* | ThermoFisher | Mm00443258_m1 |
| *IL-1B* | ThermoFisher | Mm00434228_m1 |
| *Software and Algorithms* | | |
| GraphPad Prism version 10.2.2 | GraphPad Prism | N/A |
| QuPath version 0.6.0 | QuPath | N/A |

**Abbreviations:** N/A, Not applicable; *18S*, 18S ribosomal RNA; *Axin2*, axis inhibition protein 2; *IL-1B*, interleukin 1 beta; *IL-6*, interleukin 6; *Lef1*, lymphoid enhancer-binding factor 1; *Lgr5*, leucine-rich repeat-containing G protein-coupled receptor 5; *Myc*, MYC proto-oncogene; *TNFa*, tumor necrosis factor alpha; *Wnt2b*, Wnt family member 2B.

**Supplementary Table S5. Disease Activity Index (DAI)**

| Score^a^ | Weight loss | Feces consistency | Intestinal bleeding |
| --- | --- | --- | --- |
| 0 | None | Normal | Normal |
| 1 | 0%–5% | Soft but formed | Blood traces in stool visible |
| 2 | 5%–10% | Soft but unformed | Blood traces in stool visible |
| 3 | 10%–18% | Very soft and wet | Archorrhagia |
| 4 | >18% | Diarrhea | Rectocele |

^a^The sum of the three subscores results in a combined score ranging from 0 (no changes) to 12 (severe disease activity).

**Supplementary Table S6. Histopathological Assessment**

| Score^a^ | Inflammatory infiltrates | | Epithelial changes | Ulceration |
| --- | --- | --- | --- | --- |
|  | **Severity** | **Extent** |  |  |
| 1 | Minimal | Mucosa | Minimal hyperplasia | N/A |
| 2 | Mild | Mucosa, sometimes submucosa | Hyperplasia ± goblet cell loss ± cryptitis ± erosions |  |
| 3 | Moderate | Mucosa and submucosa | Hyperplasia ± goblet cell loss ± cryptitis ± crypt abscesses | Ulcerations |
| 4 | Marked | Transmural | Hyperplasia ± goblet cell loss ± cryptitis ± multiple crypt abscesses | Extended ulcerations ± pseudo polyps |

^a^The overall histopathological score is obtained from the sum of the three subscores, ranging from 1 (no changes) to 12 (severe disease activity). **Abbreviations:** N/A, not applicable.

**
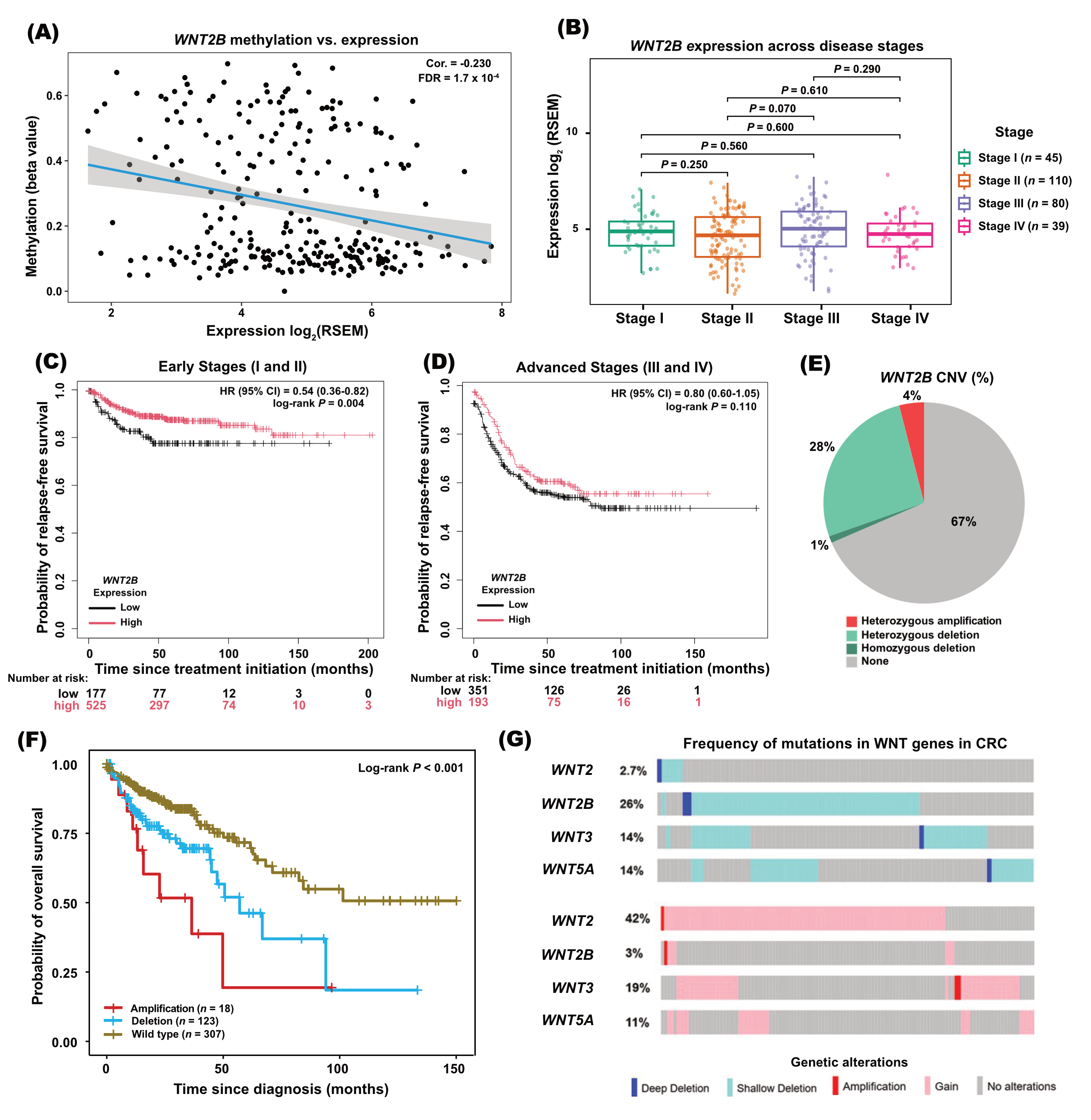
**

**Supplementary Figure S1 WNT2B Loss Predicts Worse Prognosis in Patients with Colorectal Cancer (CRC).** (**A**) Correlation between *WNT2B* expression and promoter methylation. (**B**) *WNT2B* expression levels in colon cancer samples were compared across the four disease stages. (**C-D**) Kaplan–Meier curves show the association between high (red) and low (black) *WNT2B* expression levels and relapse-free survival of patients from (**C**) early stages and (**D**) late stages, calculated by a Mantel-Cox log-rank test using Kaplan–Meier Plotter (KM Plotter). (**E**). Pie plot showing the constitution of copy number variation in the *WNT2B* gene profiled in the colorectal adenocarcinoma samples (COAD) dataset. (**F**) Kaplan–Meier curves illustrate the association between *WNT2B* mutation and patient survival, as determined by a Mantel-Cox log-rank test (*P* < 0.001). (**G**) Frequency of copy number alteration in *WNT2, WNT2B, WNT3*, and *WNT5A* gene profiles in CRC samples from the COAD dataset (*n* = 595). **Abbreviations:** CNV, copy number variation; RSEM, RNA-Seq by Expectation Maximization; cor., correlation; FDR, false discovery rate; *WNT2*, Wnt family member 2; *WNT2B*, Wnt family member 2B; *WNT3*, Wnt family member 3; WNT5A, Wnt family member 5A.

**
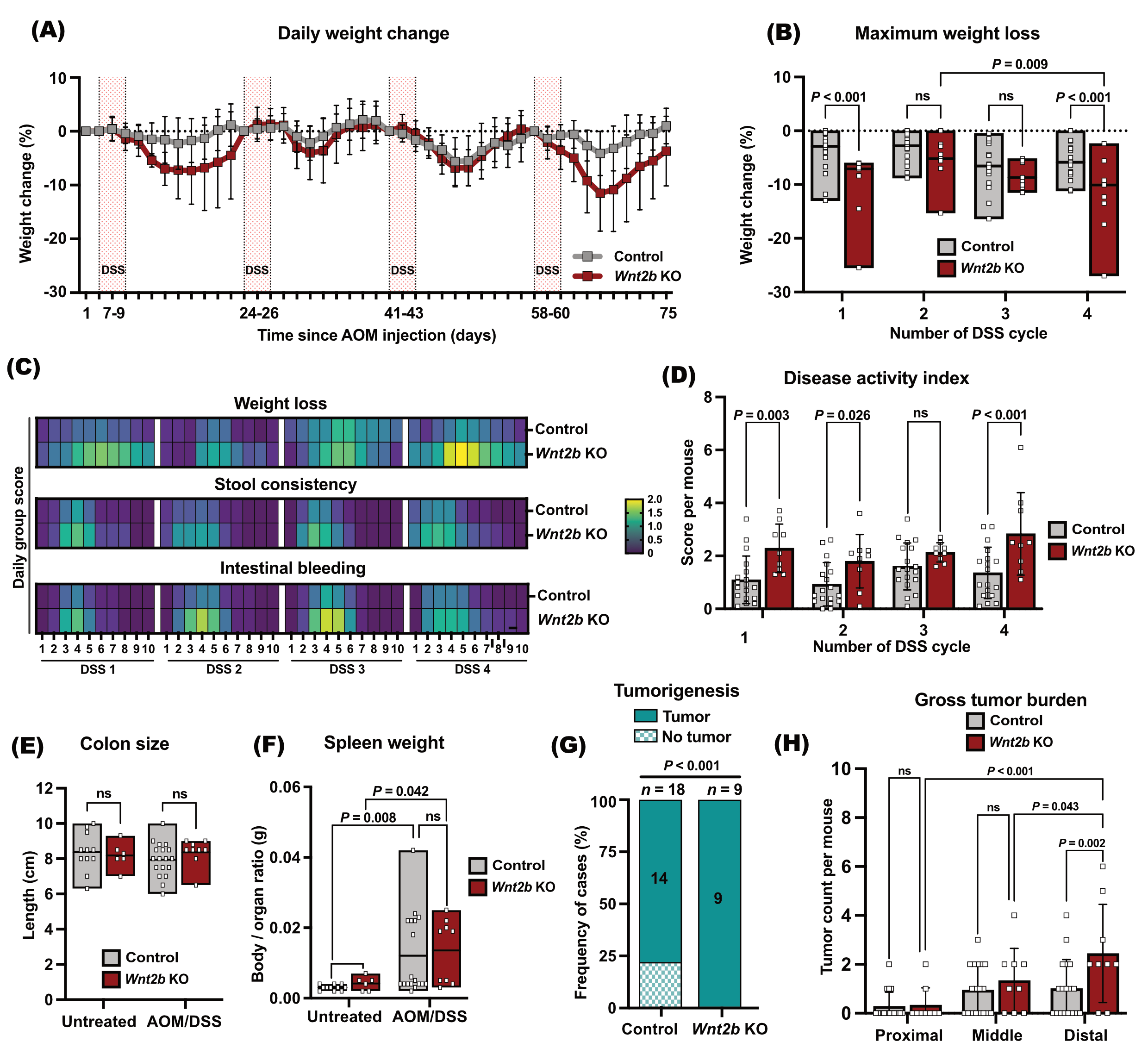
**

**Supplementary Figure S2 *Wnt2b* KO Mice Are More Susceptible to AOM/DSS-induced CAC.** (**A**) Daily group mean ± standard deviation (SD) body weight change. (**B**) Maximum body weight loss in each dextran sodium sulfate (DSS) cycle, analyzed by two-way ANOVA comparing control and *Wnt2b* KO. (**C**) Heat map showing daily mean values of weight loss, stool consistency, and intestinal bleeding during the first 10 days of each DSS cycle. (**D**) Bar graph expressing mean ± SD disease activity index during each DSS cycle, analyzed by two-way ANOVA comparing control and *Wnt2b* KO. (**E-F**) Floating bar plots expressing mean ± SD colon size (**E**) and spleen weight (**F**) per mouse, analyzed by two-way ANOVA comparing control and *Wnt2b* KO. (**G**) Frequency of animals displaying tumors during gross assessment in control (*n* = 18) and *Wnt2b* KO (*n* = 9) groups, analyzed using the chi-square test (*P* < 0.001). (**H**) Bar graphs expressing mean ± SD of regional tumor burden per mouse across different areas of the colon, analyzed by two-way ANOVA comparing control and *Wnt2b* KO mice. **Abbreviations:** ANOVA, analysis of variance; AOM, azoxymethane; CAC, colitis-associated cancer; DAI, disease activity index; DSS, dextran sodium sulfate; KO, knockout; ns, not significant (*P* > 0.05); SD, standard deviation; *Wnt2b,* Wnt family member 2b.


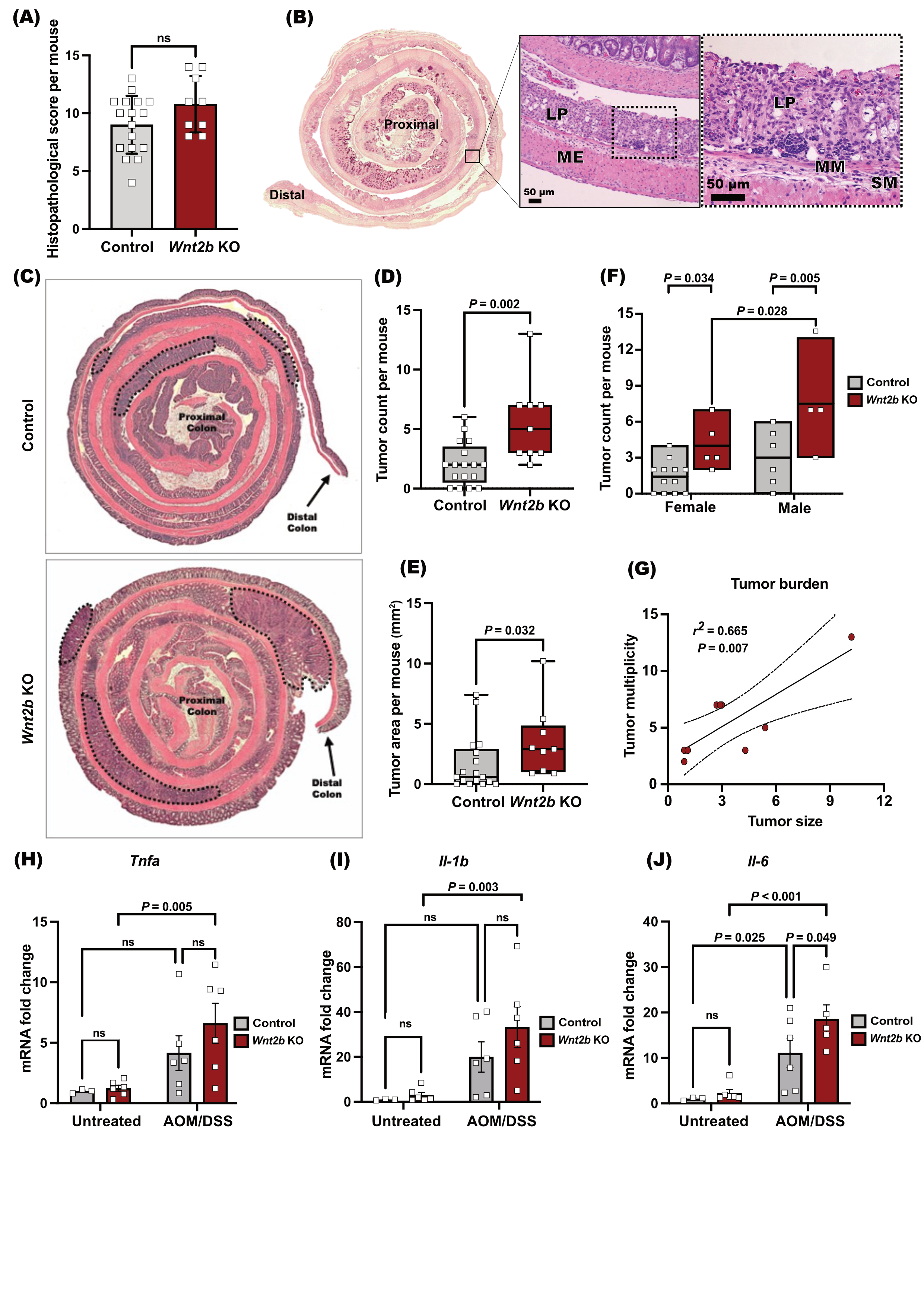


**Supplementary Figure S3 *Wnt2b* KO Mice Have Enhanced Inflammation-Driven Tumorigenesis.** (**A**) Overall histopathological scores from control and *Wnt2b* KO mice compared by a Two-tailed Student t-test (*P* = 0.095). (**B**) Representative PAS-stained Swiss-roll showing severe goblet cell depletion in *Wnt2b* KO mice that died after the first dextran sodium sulfate (DSS) cycle. Insets from a replicate H&E-stained slide highlighting epithelial lining loss and immune cell infiltration. Scale bars 50 µm. (**C**) Representative H&E-stained Swiss-rolls from control (top panel) and *Wnt2b* KO mice (bottom panel), with dotted lines highlighting tumor areas. (**D**) Box plots and whiskers expressing tumor count per mouse in control (*n* = 18) and *Wnt2b* KO (*n* = 9) groups compared by a two-tailed Mann-Whitney U test (*P* = 0.002). (**E**) Box plots and whiskers expressing average tumor area (mm^2^) per mouse in control (*n* = 18) and *Wnt2b* KO (*n* = 9) groups compared by a two-tailed Mann-Whitney U test (*P* = 0.032). (**F**) Floating bars expressing mean ± SDs comparing the sex-related differences in tumor counts via Two-way ANOVA (*P -* value shown). (**G**) Linear regression analysis of correlation between tumor size and tumor multiplicity (*r^2^* = 0.665; *P* < 0.001). (**H-J**) Graph bars expressing mean ± SDs of *Tnfa* (Tumor necrosis factor-alpha)*, Il-1b* (Interleukin 1 beta)*,* and *Il-6* (Interleukin 6) mRNA expression levels in untreated and azoxymethane/dextran sodium sulfate (AOM/DSS)-treated samples from control (*n* = 6) and *Wnt2b KO* (*n* = 6) mice assessed by qRT-PCR and compared by a Two-way ANOVA (*P*-value shown). **Abbreviations:** ANOVA, analysis of variance; AOM, azoxymethane; DSS, dextran sodium sulfate; H&E, hematoxylin and eosin; KO, knockout; LP, lamina propria; MM, muscularis mucosae; mRNA, messenger RNA; ns, not significant (*P* > 0.05); PAS, periodic acid–Schiff; qRT-PCR, quantitative reverse transcription polymerase chain reaction; SD, standard deviation; SM, submucosa; ME, muscularis externa.


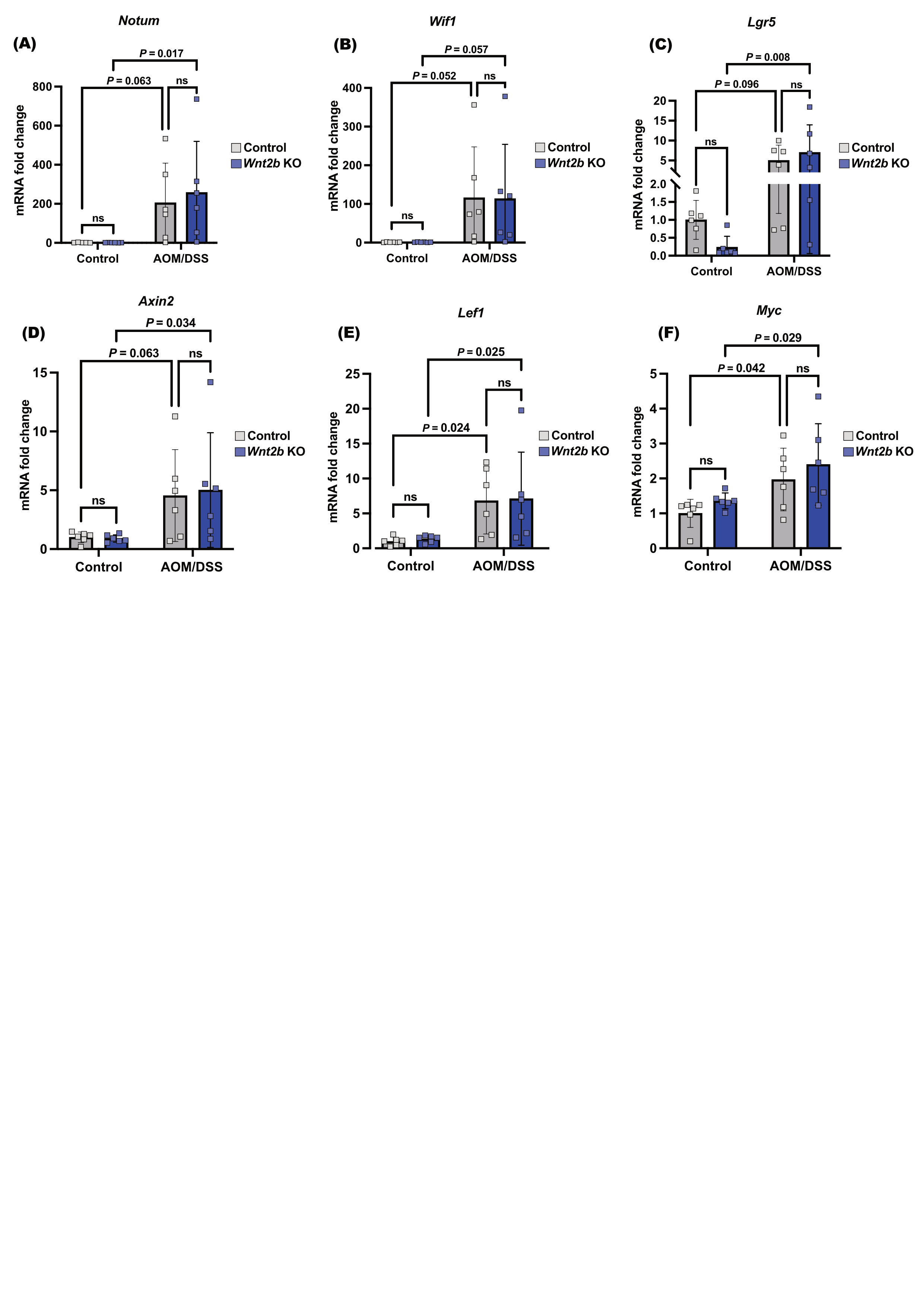


**Supplementary Figure S4 *Wnt2b KO* exhibited comparable transcript-level expression of classical β-catenin-target genes.** (**A–F**) Bar graphs showing mean ± SD of *Notum* (**A**), *Wif1* (**B**), *Lgr5* (**C**), *Axin2* (**D**), *Lef1* (**E**), and *Myc* (**F**) messenger RNA (mRNA) expression levels in untreated and azoxymethane/dextran sodium sulfate (AOM/DSS)-treated samples from control (*n* = 6) and *Wnt2b* knockout (KO) (*n* = 6) mice. Expression levels were assessed by quantitative reverse transcription polymerase chain reaction (qRT-PCR) and analyzed by two-way ANOVA (*P* > 0.05). **Abbreviations:** ANOVA, analysis of variance; AOM, azoxymethane; DSS, dextran sodium sulfate; KO, knockout; mRNA, messenger RNA; ns, not significant (*P* > 0.05); qRT-PCR, quantitative reverse transcription polymerase chain reaction; SD, standard deviation; *Wnt2b,* Wnt family member 2b.


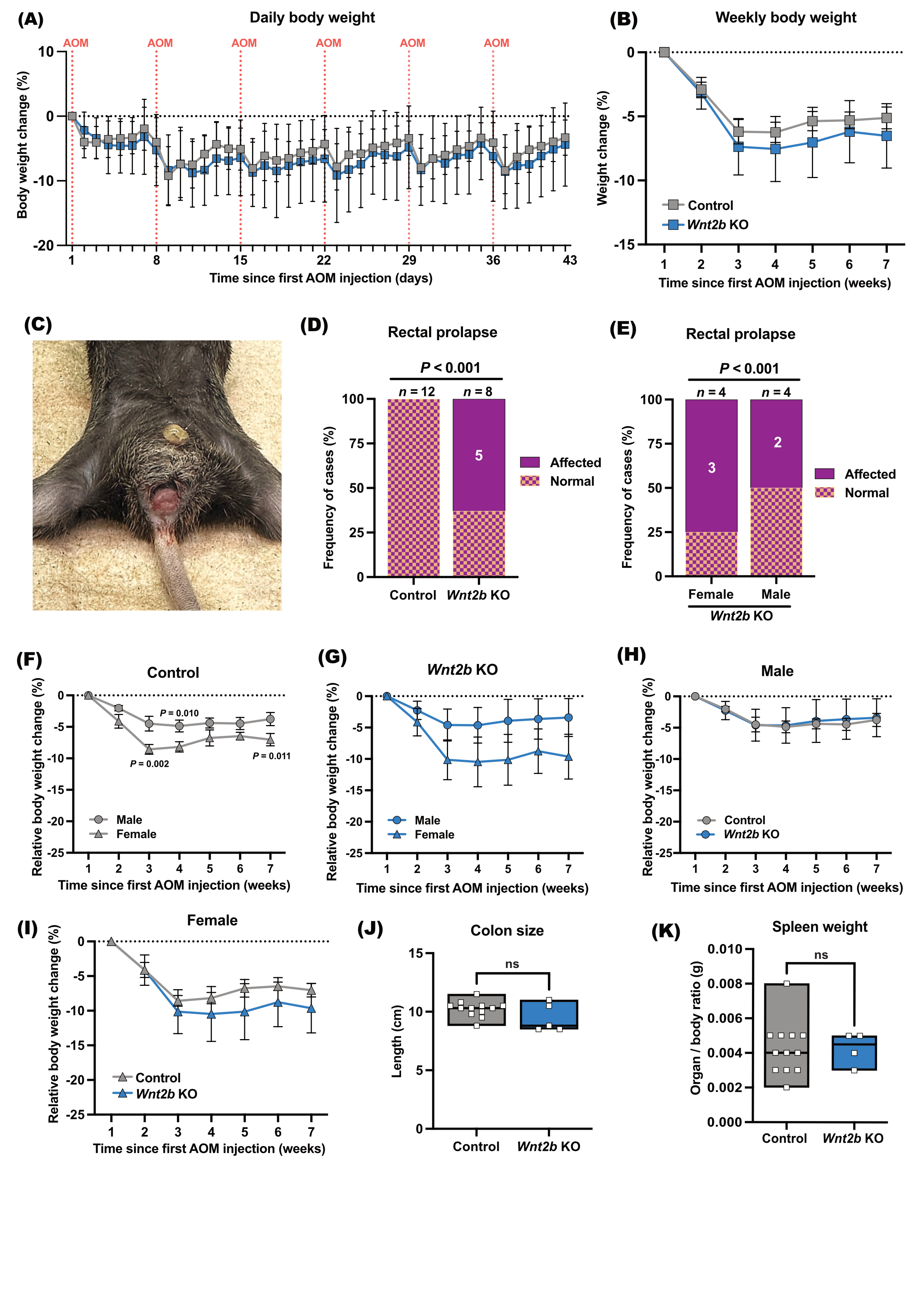


**Supplementary Figure S5 WNT2B LOF Increases Susceptibility to Sporadic Colorectal Cancer.** (**A**) Daily mean ± SD body weight change. (**B**) Weekly group mean ± SDs body weight variation, showing no significant difference between groups. (**C**) Representative picture of rectal prolapse in *Wnt2b* KO mice. (**D-E**) Frequency of rectal prolapse cases compared by group (**D**) and sex (**E**) using the Chi-square test. (**F**) Weekly body weight change in control animals. (**G**) Weekly body weight change in *Wnt2b* KO animals. (**H**) Weekly body weight change in control versus *Wnt2b* KO males. (**I**) Weekly body weight change in control versus *Wnt2b* KO females. (**J-K**) Floating bar plots expressing mean ± SD colon size (**J**) and spleen weight (**K**) per mouse, analyzed by two-tailed Mann-Whitney U test comparing control and *Wnt2b* KO mice (*P* > 0.05). **Abbreviations:** AOM, Azoxymethane; KO, knockout; ns, not significant (*P* > 0.05); SD, standard deviation; *Wnt2b*, Wnt family member 2b.


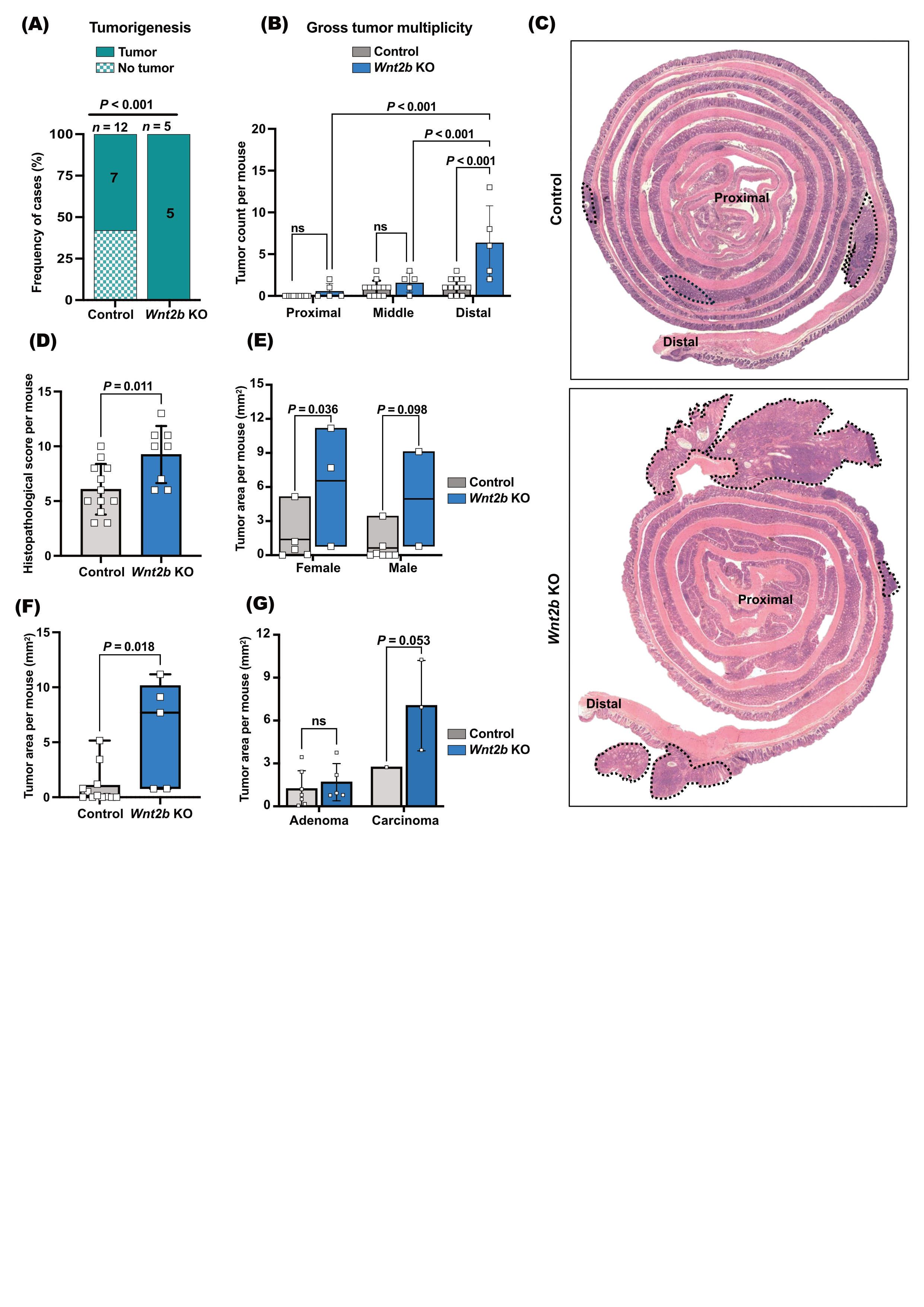


**Supplementary Figure S6 *Wnt2b* KO Mice Have Enhanced Tumorigenesis in Sporadic Colorectal Cancer Model.** (**A**) Frequency of animals presenting tumors during gross assessment in control (*n* = 12) and *Wnt2b* KO (*n* = 5) mice that survived to the end of the experiment, analyzed by a Fisher’s exact test (*P* < 0.001). (**B**) Graph bars expressing mean ± standard deviation (SD) regional tumor multiplicity per mouse across different areas of the colon, analyzed by two-way analysis of variance (ANOVA) comparing control and *Wnt2b* KO mice. (C) Representative H&E-stained Swiss-rolls from control (top) and *Wnt2b* KO mice (bottom), with dotted lines indicating tumor areas. Histopathological assessment was performed using H&E staining. (**D**) Bar graphs showing mean ± SD overall histopathological scores from control (*n* = 12) and *Wnt2b* KO (*n* = 8) mice, analyzed using a two-tailed Student t-test (*P* = 0.011). (**E**) Floating bar plots showing mean ± SD tumor measurements, comparing sex-related differences between control and *Wnt2b* KO mice using two-way ANOVA. (**F**) Box plots with whiskers showing average tumor area (mm²) per mouse in control (*n* = 12) and *Wnt2b* KO (*n* = 5) groups, analyzed using a two-tailed Mann–Whitney U test (*P* = 0.018). (**G**) Bar graphs showing mean ± SD total adenoma and adenocarcinoma areas in control (*n* = 12) and *Wnt2b* KO (*n* = 5) mice, analyzed using one-way ANOVA (*P* - value shown). **Abbreviations:** ANOVA, analysis of variance; H&E, hematoxylin and eosin; KO, knockout; ns, not significant (*P* > 0.05); SD, standard deviation; *Wnt2b*, Wnt family member 2b.

**
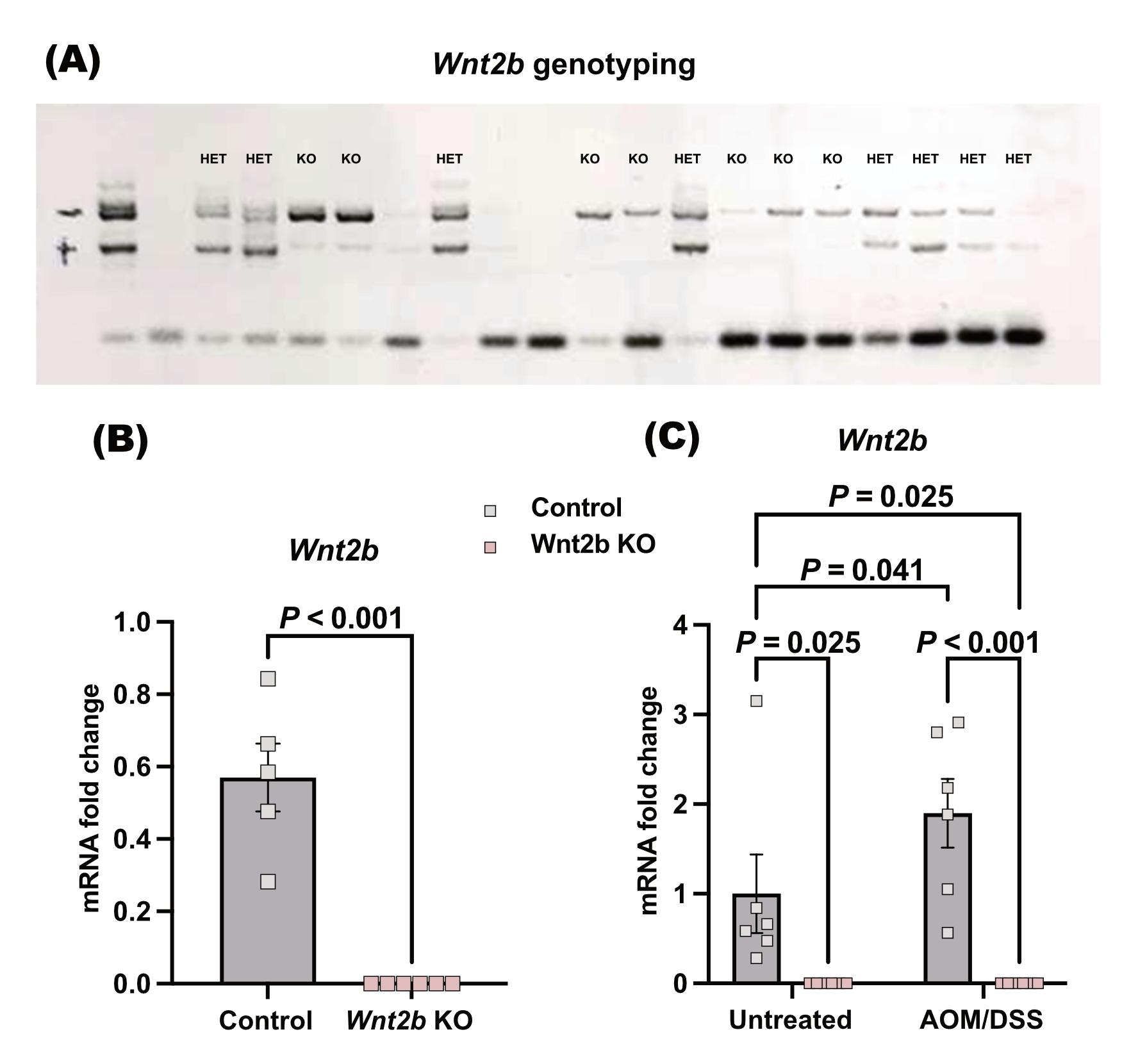
**

**Supplementary Figure S7 Validation of *Wnt2b* Deletion. (A)** Representative *Wnt2b* genotyping by agarose gel electrophoresis. Agarose gel bands represent PCR-amplified genomic DNA used for mouse genotyping, in which the upper band denotes the knockout-first allele and the lower band denotes the wild-type allele; the presence of both bands indicates heterozygosity. (**B- C**) Bar graphs showing mean ± SD *Wnt2b* mRNA expression levels in colon samples, assessed by qRT-PCR. (**B**) *Wnt2b* expression in untreated control (*n* = 6) and *Wnt2b* knockout (KO) (*n* = 6) mice, analyzed using a two-tailed unpaired Student’s t-test (*P* < 0.001). (**C**) *Wnt2b* expression in untreated and azoxymethane/dextran sodium sulfate (AOM/DSS)-treated samples from control (*n* = 6) and *Wnt2b* KO (*n* = 6) mice, analyzed by two-way ANOVA. **Abbreviations:** ANOVA, analysis of variance; AOM, azoxymethane; DSS, dextran sodium sulfate; HET, heterozygous; KO, knockout; mRNA, messenger RNA; qRT-PCR, quantitative reverse transcription polymerase chain reaction; SD, standard deviation; *Wnt2b,* Wnt family member 2b.

References

1. O'Connell AE, Raveenthiraraj S, Oliveira LFS, Adegboye C, Dasuri VS, Qi W, et al. WNT2B Deficiency Causes Enhanced Susceptibility to Colitis Due to Increased Inflammatory Cytokine Production. Cell Mol Gastroenterol Hepatol. 2024;18(2):101349.

2. Borges KS, Little DW, 3rd, Magalhães TA, Ribeiro C, Dumontet T, Lapensee C, et al. Non-canonical Wnt signaling triggered by WNT2B drives adrenal aldosterone production. bioRxiv. 2024.

3. Tsukiyama T, Yamaguchi TP. Mice lacking Wnt2b are viable and display a postnatal olfactory bulb phenotype. Neurosci Lett. 2012;512(1):48–52.

4. De Robertis M, Massi E, Poeta ML, Carotti S, Morini S, Cecchetelli L, et al. The AOM/DSS murine model for the study of colon carcinogenesis: From pathways to diagnosis and therapy studies. J Carcinog. 2011;10:9.

5. Sun W, Gao J, Yang B, Chen X, Kang N, Liu W. Protocol for colitis-associated colorectal cancer murine model induced by AOM and DSS. STAR Protoc. 2023;4(1):102105.

6. Barderas R, Villar-Vázquez R, Fernández-Aceñero MJ, Babel I, Peláez-García A, Torres S, et al. Sporadic colon cancer murine models demonstrate the value of autoantibody detection for preclinical cancer diagnosis. Sci Rep. 2013;3:2938.

7. Gobert AP, Smith TM, Latour YL, Asim M, Barry DP, Allaman MM, et al. Hypusination Maintains Intestinal Homeostasis and Prevents Colitis and Carcinogenesis by Enhancing Aldehyde Detoxification. Gastroenterology. 2023;165(3):656–69.e8.

8. Erben U, Loddenkemper C, Doerfel K, Spieckermann S, Haller D, Heimesaat MM, et al. A guide to histomorphological evaluation of intestinal inflammation in mouse models. Int J Clin Exp Pathol. 2014;7(8):4557–76.

9. Erben U, Loddenkemper C, Spieckermann S, Heimesaat MM, Siegmund B, Kuehl AA. Histomorphology of intestinal inflammation in inflammatory bowel diseases (IBD) mouse models and its relevance for IBD in men. Int J Clin Exp Pathol. 2016;9(2):408–42.

10. Bankhead P, Loughrey MB, Fernández JA, Dombrowski Y, McArt DG, Dunne PD, et al. QuPath: Open source software for digital pathology image analysis. Scientific Reports. 2017;7(1):16878.

11. He M, He B, Weng J, Cheng JQ, Gu H. Manual and Semi-Automated Measurement and Calculation of Osteosarcoma Treatment Effect Using Whole Slide Image and Qupath. Pediatr Dev Pathol. 2024;27(1):32–8.

12. Cerami E, Gao J, Dogrusoz U, Gross BE, Sumer SO, Aksoy BA, et al. The cBio cancer genomics portal: an open platform for exploring multidimensional cancer genomics data. Cancer Discov. 2012;2(5):401–4.

13. Bartha Á, Győrffy B. TNMplot.com: A Web Tool for the Comparison of Gene Expression in Normal, Tumor and Metastatic Tissues. Int J Mol Sci. 2021;22(5).

14. Tang Z, Li C, Kang B, Gao G, Li C, Zhang Z. GEPIA: a web server for cancer and normal gene expression profiling and interactive analyses. Nucleic Acids Research. 2017;45(W1):W98–W102.

15. Győrffy B. Integrated analysis of public datasets for the discovery and validation of survival-associated genes in solid tumors. Innovation (Camb). 2024;5(3):100625.

16. Liu CJ, Hu FF, Xie GY, Miao YR, Li XW, Zeng Y, et al. GSCA: an integrated platform for gene set cancer analysis at genomic, pharmacogenomic and immunogenomic levels. Brief Bioinform. 2023;24(1).
